# Infants’ reorienting efficiency depends on parental autistic traits and predicts future socio-communicative behaviors

**DOI:** 10.1093/cercor/bhae089

**Published:** 2024-05-02

**Authors:** Luca Ronconi, Chiara Cantiani, Valentina Riva, Laura Franchin, Roberta Bettoni, Simone Gori, Herman Bulf, Eloisa Valenza, Andrea Facoetti

**Affiliations:** School of Psychology, Vita-Salute San Raffaele University, Via Olgettina, 58, 20132 Milan, Italy; Division of Neuroscience, IRCCS San Raffaele Scientific Institute, Via Olgettina, 60, 20132 Milan, Italy; Child Psychopathology Unit, Scientific Institute, IRCCS Eugenio Medea, Via Don Luigi Monza, 20, 23842 Lecco, Italy; Child Psychopathology Unit, Scientific Institute, IRCCS Eugenio Medea, Via Don Luigi Monza, 20, 23842 Lecco, Italy; Department of Psychology and Cognitive Science, University of Trento, Corso Bettini, 84, 38068 Rovereto, Italy; Department of Psychology, Università degli Studi di Milano-Bicocca, Piazza dell'Ateneo Nuovo, 1, 20126 Milano, Italy; Department of Human and Social Sciences, University of Bergamo, Piazzale Sant'Agostino, 2, 24129 Bergamo, Italy; Department of Psychology, Università degli Studi di Milano-Bicocca, Piazza dell'Ateneo Nuovo, 1, 20126 Milano, Italy; Department of Developmental and Social Psychology, Via Venezia 8, University of Padova, 35131 Padova, Italy; Developmental and Cognitive Neuroscience Lab, Department of General Psychology, Via Venezia 8, University of Padova, 35131 Padova, Italy

**Keywords:** ventral attention network, salience processing, broader autism phenotype, eye movements, social interaction, communication

## Abstract

Attentional reorienting is dysfunctional not only in children with autism spectrum disorder (ASD), but also in infants who will develop ASD, thus constituting a potential causal factor of future social interaction and communication abilities. Following the research domain criteria framework, we hypothesized that the presence of subclinical autistic traits in parents should lead to atypical infants’ attentional reorienting, which in turn should impact on their future socio-communication behavior in toddlerhood. During an attentional cueing task, we measured the saccadic latencies in a large sample (total enrolled *n* = 89; final sample *n* = 71) of 8-month-old infants from the general population as a proxy for their stimulus-driven attention. Infants were grouped in a high parental traits (HPT; *n* = 23) or in a low parental traits (LPT; *n* = 48) group, according to the degree of autistic traits self-reported by their parents. Infants (*n* = 33) were then longitudinally followed to test their socio-communicative behaviors at 21 months. Results show a sluggish reorienting system, which was a longitudinal predictor of future socio-communicative skills at 21 months. Our combined transgenerational and longitudinal findings suggest that the early functionality of the stimulus-driven attentional network—redirecting attention from one event to another—could be directly connected to future social and communication development.

## Introduction

Despite the longstanding view that basic skills in the rapid attentional (re)orienting of visual attention, even in the absence of social cues, constitutes a milestone for typical social development (Conte and Richards 2021; Hendry et al. 2019; Rueda and Conejero 2020), there are very few studies in the literature precisely designed to test such a link between lower-order attentional skills and higher-order social interaction and communication skills in the typical development. Seminal findings showed that the degree to which attention is redirect by changes in the visual environment can affect joint attention abilities in typically developing infants (Butterworth and Jarrett 1991; Hood et al. 1998; Bedford et al. 2012; Parsons et al. 2019).

The orienting response involves the coordinated action of a right hemisphere dominant ventral frontoparietal stimulus-driven attention network that resets ongoing activity, also referred to as the salience network (Menon 2011; Uddin 2015), and a dorsal frontoparietal goal–directed attention network specialized for selecting and linking stimuli and responses (Corbetta and Shulman 2002; Corbetta et al. 2008). Specifically, it has been proposed that the ventral/salience network would be fundamental for detecting, integrating, and filtering salient events and, at the same time, would be a hub for mediating interactions between large-scale networks involved in externally (e.g. dorsal attentional network and central executive network) and internally (e.g. default mode network) oriented attention (Uddin and Menon 2009; Menon 2011).

From the perspective of atypical development, in the last three decades, a large body of evidence has described attentional abnormalities in autism spectrum disorder (ASD), highlighting both the dysfunctional nature of some attentional mechanisms (Ames and Fletcher-Watson 2010; Keehn et al. 2013; Sacrey et al. 2014; Federici et al. 2020) and the superiorities that these might promote in the analysis of sensory information (Dakin and Frith 2005; Robertson and Baron-Cohen 2017; Ronconi et al. 2018b; Ronconi et al. 2020). Attentional abnormalities have been associated with ASD since its first description by Leo Kanner: “*There are times, more often than not, in which she is completely oblivious to all but her immediate focus of attention.*” (Kanner 1943, p. 231). From this description, it was already evident that many patients with ASD appear to focus their attention intensely only on some element of the environment while ignoring surrounding contextual information, leading to an attitude referred to as “stimulus overselectivity” in seminal experimental studies (Schreibman and Lovaas 1973). This overselectivity was hypothesized to be the main factor leading to the difficulty of children with ASD in forming meaningful social relationships (Lovaas et al. 1979). Importantly, atypical attentional orienting has been shown in infants at elevated likelihood of developing ASD, who are infants with an older sibling diagnosed with ASD, confirming basic (nonsocial) attentional anomalies seems to be one of the earliest characteristics that distinguish those infants who later receive an ASD diagnosis from those who follow a typical developmental trajectory (Bryson et al. 2018; Elsabbagh et al. 2013; Nyström et al. 2019; Zwaigenbaum et al. 2005; Gliga et al. 2015).

Among the most robust and reproducible findings documented in ASD is the early anomalies in the rapid orienting of visual attention to information appearing far from the current locus of attention or the current point of fixation. Orienting abilities in ASD have been measured using various spatial cueing paradigms derived from the original work of Posner (1980), where one can compare response latencies to target at cued versus uncued location.

Attentional orienting involves also the ability to disengage attention from a location where attention was previously captured (i.e. reorienting) in order to shift and redirect the focus of attention onto a new location or object. Reorienting efficiency in ASD has been tested especially by examining saccadic responses in the “gap-overlap” paradigm (Kingstone and Klein 1993). Landry and Bryson (2004) examined the reorienting ability in children with ASD, and two groups of age-matched children with Down’s syndrome or with typical development; they found that the ASD group showed significantly increased latencies to reorient visual attention (on overlap trials) compared to both comparison groups. Additionally, the authors report that the frequency of fast attentional shifts (i.e. with latencies between 100 and 300 ms) for the gap condition was significantly reduced in the ASD group, suggesting that in addition to difficulty in reorient attention on overlap trials, children with ASD did not efficiently shift attention to the target, even when the central stimulus was removed. Reorienting inefficiency has been later confirmed also in low-functioning participants with ASD (Wainwright-Sharp and Bryson 1993; Courchesne et al. 1994; Kawakubo et al. 2007) and, importantly, in infants at elevated likelihood of developing ASD (Elsabbagh et al. 2013; Zwaigenbaum et al. 2005; Bryson et al. 2018). Townsend et al. (1996) found slower reorienting in adults with ASD compared to typical individuals, a finding that has been confirmed in more recent studies also in children with ASD mainly using large cues (Ronconi, et al. 2018a). In addition, some studies report that stimulus-driven (or automatic/exogenous) orienting seems to be more impaired than voluntary/endogenous (or goal-directed) shifts of attention (Ristic et al. 2005; Renner et al. 2006; Grubb et al. 2013).

Neuroimaging findings exploring the potential neural basis of such attentional reorienting problems are scarce, but some studies examining resting-state networks have reported an atypical patterns of ventral/salience network activation and connectivity (e.g. in the right anterior insula) in ASD, suggesting that among the multiple functions subserved by this network, there would be attention and processing of salient social stimuli (Odriozola et al. 2016). Similarly, the resting-state connectivity measured in the direction from the ventral attention to the salience and executive networks has been found to be significantly weaker in the ASD population (Bernas et al. 2018; see Uddin and Menon 2009).

Following the research domain criteria framework of the National Institute of Mental Health, it is interesting to corroborate whether early attentional reorienting anomalies can be considered an endophenotype of poor social-communicative skills by testing infants while considering the degree of autistic-like traits manifested by their parents. This rationale is motivated by the notion of broader autism phenotype, which considered ASD as the highest point of a range of characteristics that may exist along a continuum in the general population (Dawson et al. 2002). In fact, in most studies, infant siblings with and without later ASD are compared with infants with no family history of autism. Although this study design has been a powerful approach, the generalizability of findings from this selected subgroup to the broader population of individuals with autism and typical development remains to be established (Dawson et al. 2022). Accordingly, sensory and cognitive dysfunctions related to autism are present not only in those individuals who have been diagnosed with ASD but also in their biological relatives (Dawson et al. 2005; Belmonte et al. 2010; Sucksmith et al. 2011), who often experience milder forms of social and communication impairments similar to those observed in ASD. Research examining autistic traits has discovered that individuals with a family history of ASD tend to score higher on measures of autism (Bishop et al. 2004) and that children with parents who exhibit high but not clinically significant levels of autistic traits are more likely to display autistic traits themselves (Constantino and Todd 2005). Higher levels of autistic traits in preschool-age children predict later delays in cognitive and language functioning (Möricke et al. 2019). Finally, infants whose fathers have high autistic traits show atypical visual attention abilities (Ronconi et al. 2014; Jones et al. 2017), alpha-band brain oscillations (Riva et al. 2019), and atypicalities in visual statistical learning skills (Roberta et al. 2021), suggesting the presence of an association between parental autistic traits and cognitive functioning in their offspring.

Therefore, considering infants attentional skills in relationship with ASD traits exhibited by their parents could be additional evidence in favor of the idea that early attentional reorienting anomalies, specifically sluggish attentional reorienting, can be considered an endophenotype of poor social-communicative skills emerging at later developmental stages, not only within but also outside the boundaries defined by diagnostic criteria of ASD. To this aim, in the present study, we tested a sample of 8-month-old infants in a classic attentional cueing task, and by means of saccadic latencies, we estimated the efficiency of their rapid attentional reorienting at this early age. We included two cue–target intervals, hypothesizing that orienting efficiency would have been reduced especially at the shorter cue–target interval in infants born from parents with high subclinical ASD traits, in agreement with previous findings reviewed above showing a sluggish orienting or disengagement of spatial attention both in children with ASD and in infants at elevated likelihood of developing ASD. In a longitudinal design, we followed these infants up to 21 months to measure their competence in the social interaction and communication domains.

## Methods

### Participants

The initial sample comprised 89 infants; 18 infants were excluded because of uninterpretable eye-movement data resulting from poor calibration of the point of gaze (*n* = 2) or general fussiness (*n* = 16). The final sample comprises 71 8-month-old infants (33 females, mean age = 251.6 days, SD = 8.7, range, 232 to 280). Inclusion criteria were as follows: (i) Gestational age was ≥36 weeks; (ii) birth weight was ≥2,500 g; (iii) absence of known medical, genetic, or neurological conditions; and (iv) absence of major complications in pregnancy and/or delivery likely to affect brain development. Infants were recruited from two baby labs (IRCCS Eugenio Medea, Bosisio Parini, Italy: *n* = 41, 18 females; Department of Developmental and Social Psychology, University of Padova, *n* = 30, 14 females), with the same procedures and methods: they were recruited from a database of new parents, and parents were contacted by letter and telephone. Only in one of the two baby labs (IRCCS Eugenio Medea), follow-up information on ASD-related traits was collected in 33 children at about 21 months (M = 20.75 months; SD = 0.69).

A priori power calculations were conducted using GPower (Erdfelder et al. 1996) to estimate the smallest sample size needed to detect a medium effect size with 95% statistical power. A medium effect size was hypothesized based on previous studies on attentional disengagement anomalies in children with ASD and in high-risk infants (Ronconi et al. 2018a; Elsabbagh et al. 2013). The analysis was modeled for a repeated-measure mixed ANOVA (two groups for two measurements), and alpha equal to 0.05. Under these assumptions, the minimum total sample predicted to be needed was 54 subjects.

No differences in participant characteristics emerged between the two recruiting labs [sex, *X^2^*_(71)_ = 0.001, *P* = 0.978; age in days, *t*_(69)_ = 0.702, *P* = 0.485]. Infants were tested only after their parents gave informed consent. The ethical committee at both the IRCCS Eugenio Medea and at the Department of Developmental and Social Psychology of the University of Padova approved the present study, and the research was conducted in accordance with the ethical standards of the Declaration of Helsinki.

### Infants’ overt attentional orienting (spatial cueing) task—apparatus

The stimuli were presented with E-Prime 2.0 software on an Liquid Crystal Display (LCD) monitor. The first baby lab collected data (*n* = 30) with a pan-tilt, remote infrared eye-tracking camera (Model 504, Applied Science Laboratory, Bedford, MA) using bright pupil technology that was placed directly below the stimulus screen to record participants’ eye movements at 50 Hz. The second baby lab collected data (*n* = 41) using a Tobii ×50 eye-tracker, collecting eye data again at a rate of 50 Hz. For both baby labs, to coordinate eye movement data with respective stimulus displays, the stimulus-generating computer sent unique, time-stamped numerical codes via parallel port to the data-collecting computer, indicating the onset and type of stimulus display. Both baby labs showed stimuli on a 19-inch LCD monitor with resolution 1024 × 768 pixels. Fixation locations and changes in locations of the eye were calculated in relation to the centroids of the pupils and the corneal reflections.

### Infants’ overt attentional orienting (spatial cueing) task—stimuli and procedure

Participants were seated in a dimly lit and sound attenuated chamber. The infants sat in an infant car seat, 60 cm from the stimulus monitor. The caregiver seated on a chair behind the child and was instructed to remain in silence during the whole session. Before experimental trials began, the stimulus monitor presented animated cartoons (accompanied by a sound) at a minimum of five (up to nine) different locations to calibrate the eye tracker. The calibration process typically took 5 min. All subsequent eye data were calculated from these calibration values.

**Fig. 1:**
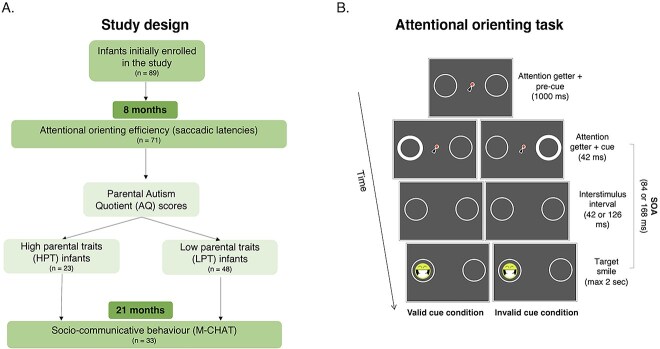
Schematic representation of the transgenerational and longitudinal study design A) and of the experimental procedures of the attentional (re)orienting task B).

After the calibration phase, an attention getter (animated cartoon) was presented on the center of the screen to direct the infant’s gaze. Four different attention-getters, randomly presented across the trials, were chosen to capture and sustain infants’ attention. As soon as the infant looked at the central attention getter for 300 ms, two colored circles (6° of diameter) were automatically presented on a black background on the left and right side of the screen (11° of eccentricity from the center, with the two edges of the circles separated by 16°) (Fig. 1). The circles varied in colors (red, green, yellow, and blue) randomly across different trials. A cue, the thickening of one of the two circles (from 0.2° to 0.7°), then appeared for 42 ms, in addition to the cartoon. This brief change did not let the infant orient eye movement toward the cue, thus being ideal to measure covert attention (Richards 2001). Moreover, the presentation of the cue at the same time as the dynamic central fixation stimulus helped prevent saccades to the peripheral cue (Johnson and Tucker 1996). Finally, a visual target, consisting in a smiling and flickering schematic face (3.2, flickering at one cycle of 168 ms, 64 ms on–64 ms off, 5.95 Hz), appeared after one of two intervals (84 or 168 ms). Four different target types were randomly presented across trials to sustain infants’ attention. The target remained visible until the participant looked at it or for a maximum of 2 s. This terminated the trial, and another trial began at the central attention-getter. Valid cues (the cue was presented in the same circle that includes the target), neutral cues (consisting in the thickening of both circles, providing no information on the target location), or invalid cues (the cue was presented in the circle that did not include the target) were randomly intermixed. Sixty trials divided into three blocks were presented to each infant. Each block consisted of 8 valid, 8 invalid, and 4 neutral trials. Thus, for a total of 24 valid trials [12 for each cue–target stimulus onset asynchrony (SOA)], 24 invalid trials (12 for each cue–target SOA), and 12 neutral trials (6 for each cue–target SOA) were presented to each infant. Neutral cue trials have been included in half of the trials to maximize the attentional orienting toward one visual hemifield induced by valid and invalid cues and to avoid infants developing habituation toward a lateralized cue presentation. However, analyses will focus only on valid and invalid trials.

### Data analysis

The display was virtually divided into three squared areas of interest (AOIs): one surrounded the position of the central attention-getter, and two corresponded to the two circles. Each AOI measured approximately 7.8° on each side. Time to target fixation (TTF) was a dependent variable (fixation threshold settings: duration > 100 ms, maximum displacement < 1° of visual angle). As an important step to perform final statistical analyses (Wass et al. 2014), we checked that the total number of usable trials were on average 32 (SD = 9.3) out of the 48 trials presented, and the average usable trials did not differ neither between the two baby labs [*t*_(69)_ = −1.03, *P* = 0.31], nor between the LPT and HPT groups [*t*_(69)_ = 0.46, *P* = 0.65].

### Evaluation of self-reported autistic traits in parents

Both parents of each participating infant completed the Italian version of the Autism Quotient (AQ) questionnaire (Baron-Cohen et al. 2001). The questionnaire includes 50 items grouped in five subscales: social skills, attention switching, attention to detail, communication, and imagination. The total AQ score ranges from a minimum of 0 to a maximum of 50, and it is calculated by adding up all of the scores from the different subscales, with higher scores indicating higher autistic traits.

Similarly to recent studies (Roberta et al. 2021), infants were divided into two groups based on the total AQ score obtained by each of their parents using the broader autism phenotype cut-off of +1 SD above the standardized mean, which corresponds to 21 points for males and 20 points for females.

Infants with both parents scoring below +1 SD were included in the low parental traits (LPT) group (*n* = 48), whereas infants who had at least one parent who scored above +1 SD were included in the high parental traits (HPT) group (*n* = 23). Among the 23 HAT infants, 12 (52.2%) were included in the group based on the father’s AQ score and 11 (47.8%) were included based on the mother’s AQ score. Table 1 reported descriptive statistics in the HPT and LPT group.

**Table 1:** Socio-demographic descriptive statistics of participants.

	HPT (*n* = 23) Mean (SD)	LPT (*n* = 48) Mean (SD)	Group difference *P*-value (Cohen’s *d*, 95% C.I.)
Age (days)	256.16 (16.27)	250.17 (7.36)	0.094 (−0.54, −1.05/−0.04)
Maternal age at birth	34.09 (3.96)	34.15 (4.41)	0.173 (0.014, −0.48/0.51)
Paternal age at birth	37.36 (4.94)	37.02 (5.00)	0.960 (−0.068, −0.57/0.43)
Maternal educational level	59.57 (16.65)	66.46 (14.66)	0.791 (0.45, −0.05/0.95)
Paternal educational level	50.22 (18.74)	58.51 (20.72)	0.080 (0.41, −0.09/0.91)
SES	63.70 (18.35)	68.02 (19.64)	0.110 (0.22, −0.27/0.72)
Maternal autistic traits	17.64 (5.56)	12.52 (4.51)	0.001 (−1.08, −1.61/−0.55)
Paternal autistic traits	20.13 (7.26)	13.80 (4.29)	0.001 (−1.17, −1.70/−0.64)
M-CHAT failed item	0.7 (1.2)	0.8 (1.03)	0.602

### Follow-up assessment at 21 months old

In one of the baby labs (IRCCS Eugenio Medea), follow-up information on ASD-related traits was collected in 33 children at about 21 months (14 infants for the HPT group and 19 infants for the LPT group) using the M-CHAT questionnaire, a checklist to detect children at risk for ASD based on commonly observed child behaviors. The M-CHAT is one of the parental questionnaires most commonly used in primary care and clinical settings (Johnson et al. 2007; Robins 2008). It requires the parent to report on the presence of specific child behaviors in a checklist of 23 yes/no items (total items). The presence of abnormal behavior was assigned a score of 1 and the total score was interpreted. In addition, we considered the six items pertaining to social relatedness and communication (critical items) that were found to have the best discriminability between children diagnosed with and without ASD (Robins et al. 2014). The critical items are as follows: (i) Does your child take an interest in other children? (ii) Does your child ever use his/her index finger to point, to indicate interest in something? (iii) Does your child ever bring objects over to you (parent) to show you something? (iv) Does your child imitate you? (e.g. if you make a face, will your child imitate it?) (v) Does your child respond to his/her name when called? (vi) If you point at a toy across the room, does your child look at it?

For the purpose of this study, we included in the analyses both the total score (failed items) and the number of critical items.

## Results

### Infants’ visual attention depends on parental autistic traits: group analysis

We conducted a mixed-design ANOVA using the cuing effect (i.e. differences in saccadic reaction times (RTs) between invalid and valid conditions) as a dependent variable. We considered the SOA as the within-subject factor (2 levels: 84 vs. 168 ms), and the group as between-subjects factor (two levels: HPT vs. LPT).

**Fig. 2:**
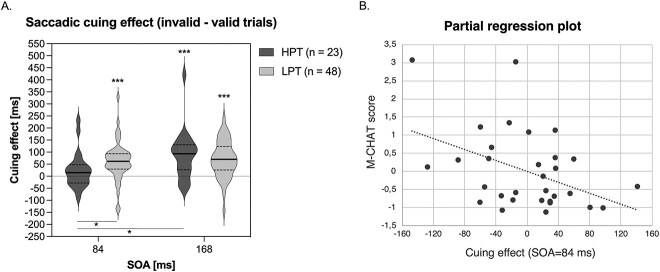
A) Violin plot showing the cuing effect (difference in saccadic latencies between the invalid and valid trials) as a function of group (HPT: high parental traits and LPT: low parental traits) and SOA. Horizontal lines represent the median (continuous line) and the first and third quartiles (dashed lines). ^*^P < 0.05, ^*^^*^^*^P < 0.001. B) Partial regression plot showing the linear relationship between the early attentional orienting abilities (SOA = 84 ms) measured at 8 months and the degree of socio-communicative problems measured at 21 months, after controlling for maternal age, paternal age, and cognitive level.

This ANOVA revealed a main effect of SOA [*F*_(1,69)_ = 7.693, *P* = 0.007, η^2^_p_ = 0.100], whereas the group main effect was not significant [*F*_(1,69)_ = 0.552, *P* = 0.46, η^2^_p_ = 0.008]. Remarkably, the ANOVA revealed a significant interaction between the SOA and the group [*F*_(1,69)_ = 4.533, *P* = 0.037, η^2^_p_ = 0.062; Fig. 2A].

This interaction was further explored with planned comparisons (i.e. one-sample and independent-samples *t*-tests) to explore both within and between groups differences. Difference in the cuing effect were observed between groups at the shorter SOA [*t*_(69)_ = −1.99, *P* = 0.025, one tail; Cohen’s d = 0.51, 95% CI = 0.001/1.01], while at the longer SOA, the cuing effect was not different [*t*_(69)_ = 0.74, *P* = 0.23, one tail; Cohen’s *d* = −0.19, 95% CI = −0.69/0.31]. In addition, the HPT group showed a significantly reduced cuing effect at the shorter as compared to the longer SOA [*t*_(22)_ = −2.53, *P* = 0.019; Cohen’s *d* = 0.86, 95% CI = 0.10/1.33], while in the LPT group the cuing effect did not differ across SOAs [*t*_(47)_ = −0.63, *P* = 0.535; Cohen’s *d* = 0.10, 95% CI = −0.29/0.51].

Finally, in the LPT group, the cuing effect was significantly different from 0 both at the shorter [*t*_(47)_ = 5.36, *P* < 0.001; Cohen’s *d* = −0.92] and longer SOA [*t*_(47)_ = 6.43, *P* < 0.001; Cohen’s *d* = −0.93]. Conversely, in the HPT group, the cuing effect was significantly different from 0 at the longer SOA [*t*_(22)_ = 4.21, *P* < 0.001; Cohen’s *d* = −0.95], but not at the shorter one [*t*_(22)_ = 1.49, *P* = 0.149; Cohen’s *d* = −0.29].

### Infants’ visual attention depends on parental autistic traits: individual data

Because a difference in cuing effect was observed between groups at the shorter SOA, we used individual data of cuing effect at the shorter SOA to index the attentional reorienting dysfunction. Nineteen out of 23 children of the HPT group (about 83%) were below the mean of the LPT group. Thus, the attentional orienting/disengagement dysfunction that we have documented in group analysis appears to be rather frequent among infants with HPTs.

### Infants visual attention predicts future socio-communicative behaviors: longitudinal partial correlation and regression analysis

To determine the possible relationship between early attentional reorienting skills (cuing effects obtained by saccadic latencies) measured at 8 months and future social interactions and communications skills measured at 21 months with the M-CHAT, we used a 2-fold approach.

First, Pearson correlations were performed between the cuing effect at 84 and 168 ms SOAs and both failed and critical items measured in the M-CHAT (for these measures, higher scores mean higher social-communicative difficulties). We found that the cuing effect at the shorter SOA (84 ms) correlated significantly and negatively with both total failed [*r*_(33)_ = −0.428, *P* = 0.013] and critical [*r*_(33)_ = −0.464, *P* = 0.007] M-CHAT items. The same correlations were not found for the cuing effect at the longer SOA (168 ms) (both *P*s > 0.427).

Second, to determine the predictive relationship between attentional reorienting and future socio-communicative skills in a more stringent way, a three-step fixed-entry multiple regression analysis was performed on the individual data of the 33 children. The dependent variable was the M-CHAT score (total failed items) and the predictors were, in the first block, (i) infants’ sex (male/female); (ii) infants’ cognitive level as assessed by the Bayley Scales of Infant Development (Bayley, 2012); (iii) maternal age; and (iv) paternal age. In the second block, we inserted the cuing effect at the longer SOA (168 ms). In the third block, we inserted the cuing effect at the shorter SOA (84 ms). Overall, the regression model accounted for 23% of the future socio-communicative skills variance. Chronological age, cognitive level, and maternal/paternal age accounted for 3.4% of the variance of socio-communicative skills [*F*-change_(4, 27)_ = 0.23, *P* = 0.92]. Similarly, the cuing effect at the longer SOA accounted only for 0.2% of the unique variance of socio-communicative skills [*F*-change_(1, 26)_ = 0.047, *P* = 0.829]. Importantly, the infants’ cuing effect at the shorter SOA entered last accounted for 19.3% of the unique variance of future social-communicative behaviors [*F*-change_(1, 25)_ = 6.27, *P* = 0.019; Fig. 2B].

## Discussion

The present study aimed to investigate the early developmental course of attentional reorienting in infants that exhibit different degrees of familial ASD-like traits. Since subthreshold cognitive and behavioral ASD-like characteristics aggregate in family members, and since attentional anomalies appear to be one of the earliest cognitive markers of children at risk for ASD (see Dawson et al. 2022 for a recent review), we wanted to test the research domain criteria hypothesis that a reduced efficiency of the attentional reorienting system (Corbetta et al. 2008) could be already present in 8-month-old infants also when their parents manifest a higher presence of ASD traits.

Our results show that the rapid reorienting of visual attention to target in response to a peripheral and unpredictive cue measured in infants at 8 months of age was related to autistic traits exhibited by their parents. While infants with low familiar traits were able to rapidly reorient their attentional focus to a peripheral location in their visual field already within ~80 ms from the cue onset, infants with high familiar traits showed a significantly delayed cuing effect, showing an efficient attentional reorienting only at the longer cue–target interval (~170 ms). Although this sluggish stimulus-driven attentional orienting could be also explained by a primary deficit of attentional disengagement from the central attentional getter, our findings suggest a primary deficit in the basic mechanisms of reorienting system controlled by the ventral frontoparietal attention (Corbetta et al. 2008) and/or salience network (Uddin 2015).

Importantly, we followed these infants longitudinally and had the chance to measure the developmental trajectory of their future social and communicative skills as could be measured at 21 months of age. This result was not only visible through simple correlational analysis, but also performing a prospective longitudinal regression analysis where we aimed to control for other important factors such as the infants’ general cognitive level measured at 8 months, their sex, their parents’ age, and sustained attention (i.e. the cuing effect at 168 ms). We further confirmed that rapid attentional reorienting (i.e. the cuing effect at 84 ms) was the only predictor of the social and communicative skills as measured by the M-CHAT at 21 months of age, and the percentage of variance in M-CHAT score predicted was particularly remarkable (about 20%), considering that infants did not have a history of ASD in their families.

These findings are consistent with other studies showing that lower-level attentional processes may significantly impact the development of higher-level cognitive and behavioral domains (for a review, see Sacrey et al. 2014). In particular, an early reorienting system dysfunction could predict future social and communication development redirecting attention from one event to another. For example, inefficient and slow attentional reorienting could lead to atypical visual exploration of the environment (Elsabbagh et al. 2009; Landry and Parker 2013), to longer times taken to process social cues that normally facilitate social interaction, thus biasing attention toward nonsocial aspects of the environment (Sasson et al. 2008, 2011). Slower attentional reorienting system might also impact emotion regulation, as it is known that shifting attention from a stimulus causing distress to a distracting stimulus can temporarily alleviate distress in infants (Harman et al. 1997; Bryson et al. 2018). Thus, early impairments in attentional reorienting system can lead to atypical emotion/arousal regulation, over-reactivity, sensory overload, and restricted temperamental styles (Bryson et al. 2004, 2007, 2018; Ronconi et al. 2018b; Ronconi et al. 2018a). Moreover, attentional reorienting might be—as largely acknowledged—responsible for typical social development (Dawson and Lewy 1989; Keehn et al. 2013; Sacrey et al. 2014; Ronconi et al. 2016). For example, ​​children who are quick to reorient from the current attentional locus showed higher indices of effective joint attention (Bryson et al. 2018; Elsabbagh et al. 2013; Schietecatte et al. 2012). The present findings are also consistent with previous research showing, with a purely correlational design, that paternal autistic traits were linked with visual attention in their offspring (Ronconi et al. 2014). In the present study, we employed a different approach, dividing participants based on the degree of autistic-like traits jointly exhibited by both their parents. Indeed, splitting participant according to maternal vs. paternal traits would have considerably undermine our statistical power (we would have included only 12 infants in the “paternal” HPT group and 11 infants in the “maternal” HPT group). Future studies employing larger samples could overcome this limitation to better study the differential contribution of maternal and paternal traits to development of early attentional skills in infancy. The same approach used here applied to a larger population could also better investigate the continuum of clinical traits. Indeed, another limitation of the present study is that the M-CHAT may not be so sensitive as instrument to detect subtle differences in socio-communicative behaviors considering that the participants of our study were children from the typically developing population. Despite the reduced sensitivity of the instrument, the results indicate that greater attentional reorienting difficulties detected at 8 months predict the development of communication and social skills at 21 months, confirming a rich and consolidated scientific literature coming from both findings in infants with future development of ASD and from studies in typically developing children. Potentially, even better results in future studies could be found with instruments that are more sensitive in the typical development.

The ventral stimulus–driven attentional network and/or salience network are fundamental to detect novel stimuli and guide attention toward the source of relevant information, as well as to integrate and filter salient information and mediate the interactions with other important large-scale (e.g. default mode, dorsal attentional, and/or central executive) networks (Corbetta et al. 2008; Uddin and Menon 2009; Menon 2011; Uddin 2015). Additionally, resting-state neuroimaging studies have recently documented an atypical pattern of activation in the ventral attentional network and/or salience network activation and its connectivity with other crucial social large-scale networks, such as the default mode network (Odriozola et al. 2016; Bernas et al. 2018).

Based on the current findings and given that the paradigm employed in the present study is a proxy for the ventral attentional network functioning (Corbetta and Shulman 2002; Corbetta et al. 2008; Corbetta and Shulmann 2011), we can speculate that the integrity of this neural network in the early stages of life could play a crucial role for the emergence of social and communication skills. Although the field of large-scale brain networks mapping in the early human development is still in its infancy, the available evidence show that resting-state networks (or their prototypes) have been largely formed after the third trimester (for a review, see Zhang et al. 2019), compatibly with the developmental pathway that we have longitudinally traced in the present study.

A primary novel contribution of this research is to show that early indicators of the core anomalies that are present in children with ASD or in infant siblings who later develop ASD can be extended to infants whose parents exhibit a high degree of autistic traits. These early deficits in the rapid attentional reorienting system may be related to later impairments in higher-level domains, such as communication skills and responses to social and nonsocial stimuli (Chawarska et al. 2013; Hutman 2013). In accordance with this, studies conducted on individuals with ASD have demonstrated that other basic visual abnormalities, including superior performance in visual search tasks (Gliga et al. 2015; Joseph et al. 2009), difficulties in biological motion processing (Federici et al. 2020), and abnormal patterns of visual fixation for social stimuli (Klin et al. 2002; Jones and Klin 2013), can predict impairments in social interaction and communication.

Currently, there is no reliable identification of robust biomarkers for ASD in infants and toddlers, possibly due to the extensive clinical and etiological heterogeneity linked with ASD (Geschwind 2011; Parellada et al. 2023). Biobehavioral traits have the advantage of being easier to measure and less expensive than molecular or neuroimaging biomarkers (Jones et al. 2017; Dawson et al. 2022). In this sense, measures of eye-tracking in a simple visual attentional paradigm are more suitable to be tested in infants before 1 year of age (Hamner and Vivanti 2019). At the same time, from both a clinical and neurobiological perspective, ASD is heterogeneous. Unusual patterns of visual preference (see Gori et al. 2016 for a review) and visual attention as indexed by eye tracking have been proven to be hallmarks, but it is still unclear if they can be used to define an early biomarker of ASD as a whole or be used even to define different subtypes (Pierce et al. 2016). To face this issue, large cohorts of children and infants are required and having a biomarker that is able to depict the onset of ASD or to detect some core features with relatively low-cost technology would be a significant advantage.

Especially after the Covid-19 pandemic, where many labs around the world were unable to conduct in-person studies, the need for searching solutions for doing methodologically appropriate research outside the typical infant laboratories has turned out to be a major challenge in the field. Remote web-based eye-tracking studies (Kominsky et al. 2021; Rhodes et al. 2020; Sheskin et al. 2020; Steffan et al. 2024) based on automated procedure have become increasingly popular in recent years and seem a particularly important accomplishment for developmental research, as they would allow testing children at home and reach large, diverse, and more representative samples of the population, to facilitate data collection across many laboratories, centers and geographical areas (Byers-Heinlein et al. 2022; Visser et al. 2022). Technological advances now allow conducting eye-tracking online with infants, as shown recently by two studies that have directly compared webcam-based eye-tracking and in-lab eye-tracking data from 4- to 6-month-old infants (Bánki et al. 2022) and from 9 to 36 months (Calignano et al. 2023). Remote web-based eye-tracking can be suitable also for implementing early trainings of visual attention in infancy (Wass et al. 2011; Goodwin et al. 2021; Perra et al. 2022).

In conclusion, in the present study, we showed that a simple measure of eye tracking obtained during a stimulus-driven attentional cueing task could be used as a reliable reorienting index for studying the precursors of higher-order social and communicative domains and to detect potentially altered neurodevelopmental pathways. Because the reorienting system functioning in infants longitudinally predicts future development of their social and communication skills, and parents’ social and communicative behavior is trans-generationally linked with this efficient reorienting mechanism, we demonstrate the direct impact that early development of reorienting system could have on future social cognition.

## Data Availability

Data are available upon requests to the Authors. Data are not publicly available due to privacy or ethical restrictions.

## Permission to reproduce material from other sources

Not applicable (no material from other sources).
